# Network Pharmacology and Molecular Docking Study on the Multi-Target Mechanisms of *Aloe vera* for Non-Alcoholic Steatohepatitis Treatment

**DOI:** 10.3390/plants11243585

**Published:** 2022-12-19

**Authors:** Tan Khanh Nguyen, Huy Hieu Phung, Won Jun Choi, Hee-Chul Ahn

**Affiliations:** College of Pharmacy, Dongguk University-Seoul, Goyang 10326, Republic of Korea

**Keywords:** non-alcoholic steatohepatitis (NASH), *Aloe vera*, molecular docking, network pharmacology, Gene Ontology (GO), Kyoto Encyclopedia of Genes and Genomes (KEGG) enrichment analyses

## Abstract

Non-alcoholic steatohepatitis (NASH) is a leading cause of chronic liver disease with limited treatment options. The widely distributed plant *Aloe vera* has shown protective effects against NASH in animals, yet the precise mechanism remains unknown. In this study, we investigated the potential mechanisms underlying the anti-NASH effects of *Aloe vera* using a network pharmacology and molecular docking approach. By searching online databases and analyzing the Gene Expression Omnibus dataset, we obtained 260 *Aloe vera*–NASH common targets. Gene Ontology and Kyoto Encyclopedia of Genes and Genomes enrichment analyses showed that the common targets were strongly associated with the key pathological processes implicated in NASH, including lipid and glucose metabolism, inflammation, apoptosis, oxidative stress, and liver fibrosis. Four core proteins, AKT serine/threonine kinase 1 (AKT1), tumor necrosis factor alpha (TNFα), transcription factor c-Jun, and tumor suppressor protein p53, were identified from compound–target–pathway and protein–protein interaction networks. Molecular docking analysis verified that the active ingredients of *Aloe vera* were able to interact with the core proteins, especially AKT1 and TNFα. The results demonstrate the multi-compound, multi-target, and multi-pathway mechanisms of *Aloe vera* against NASH. Our study has shown the scientific basis for further experiments in terms of the mechanism to develop *Aloe vera*-based natural products as complementary treatments for NASH. Furthermore, it identifies novel drug candidates based on the structures of *Aloe vera*’s active compounds.

## 1. Introduction

With the explosion of overnutrition and a sedentary lifestyle, non-alcoholic fatty liver disease (NAFLD) and its advanced form, non-alcoholic steatohepatitis (NASH), have become global health problems. NAFLD is a spectrum of conditions in which lipids accumulate in more than 5% of the hepatocytes (i.e., liver steatosis) without alcohol abuse [1]. Furthermore, NAFLD is considered the hepatic manifestation of metabolic syndrome owing to its strong association with insulin resistance and other metabolic disorders [2]. While most patients with NAFLD are asymptomatic, 20–30% eventually develop NASH, in which steatosis is accompanied by liver injury, inflammation, and fibrosis [3]. Patients with NASH face increased risks of hepatocellular carcinoma (HCC), the need for liver transplantation, and liver-related mortality [4,5]. An effective preventive strategy for NASH is currently lacking; despite intensive research, no therapy has been approved. Weight loss (≥7% of total body weight) through diet and exercise is the primary goal for NASH [1]. However, most patients cannot achieve this due to poor adherence and inadequate healthcare support [6]. NASH pathogenesis involves multiple parallel hits, including apoptosis, oxidative stress, and adipokines [7], which present a challenge for the conventional one drug–one target strategy. In this context, natural products (NPs) have been shown to be promising because they contain various bioactive components that act on numerous targets, thereby affecting multiple pathological processes. Many diverse herbs and NPs are currently used as complementary treatments for NASH [8].

*Aloe vera* belongs to the Liliaceae family. Originating in Africa, this plant is now grown worldwide. *Aloe vera* is famous for its enormous health and nutritional benefits and has long been used in food, drinks, and cosmetics. Moreover, *Aloe vera* is present in many herbal remedies thanks to its numerous attractive biological features. For example, *Aloe vera* leaves contain a waxy gel with cooling and antibacterial properties that can relieve acne, pimples, skin burns, and injuries [9]. It is also used for metabolic disorders because of its ability to control glucose and lipid metabolism [10]. In addition, *Aloe vera* possesses antitumor properties and is a component of several herbal remedies for cancer [11]. Recently, *Aloe vera* has been reported to exert anti-inflammatory and antioxidant effects on NASH in vivo [12]; however, the precise mechanisms remain unclear. Therefore, the potential findings about target proteins of *Aloe vera* in the treatment of NASH will be evidence for in vitro and in vivo studies to evaluate the activity and safety in the use of *Aloe vera* and its components for clinical treatment.

Since the beginning of civilization, plants have provided constant support for food, medicine, and other essentials. Developing NPs into medicines is a promising research direction. However, studying the mechanisms of NP-based drugs using conventional pharmacological and biochemical methods is challenging because of their complex composition and diverse disease targets. Therefore, network pharmacology has emerged as a revolutionary field that integrates systems biology and bioinformatics to explore the molecular mechanisms of NPs. By studying the interactions between active ingredients and disease targets, bioinformatic methods and network-pharmacology-based research provides a better understanding of the combinatorial rules of herbal remedies, helping clinical practitioners design herbal formulae rationally [13]. In this study, we applied network pharmacology and molecular docking methods to explore the molecular mechanisms of *Aloe vera* for NASH treatment. Our study provides a theoretical basis for studying and developing *Aloe vera*-based drugs that are effective against NASH.

## 2. Results

### 2.1. Active Ingredients of Aloe vera

A total of 53 components extracted from parts of *Aloe vera* were retrieved from the traditional Chinese medicine systems pharmacology database and analysis platform (TCMSP) database, among which eight ingredients satisfied the selection criteria of oral bioavailability (OB) ≥ 30% and drug likeness (DL) ≥ 0.18. Additionally, the anthraquinone glycoside aloin was included because it is a major constitute of *Aloe vera* [14], possesses a high DL value (0.71), and has shown protective effects against NASH in vivo [15]. Moreover, glycosides can be converted to aglycones in the intestine, which may show improved OB compared with the original form. The final active ingredient list of *Aloe vera* consisted of nine compounds (Table 1).

### 2.2. Common Targets of Aloe vera and NASH

**Figure 1:**
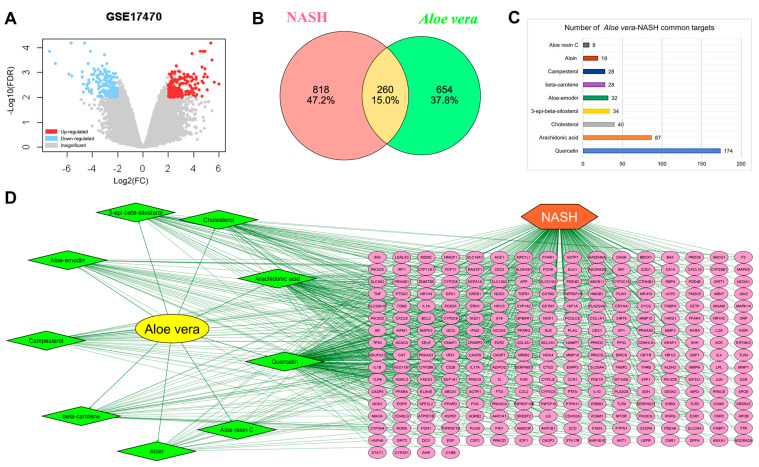
Common *Aloe vera* and non-alcoholic steatohepatitis (NASH) targets. (**A**) Volcano plot of differentially expressed genes from the GSE17470 dataset. Red and blue dots represent upregulated and downregulated genes, respectively; gray dots indicate insignificant changes. (**B**) Intersection of the *Aloe vera* and NASH-related targets. Red and green circles represent the predicted targets of NASH and *Aloe vera*, respectively. The yellow area reflects the common targets. (**C**) Number of common targets of the active ingredients. (**D**) *Aloe vera* and NASH herb–component–target-disease network. Green nodes display the active ingredients; pink nodes represent *Aloe vera*–NASH common targets, and edges reflect the interactions between nodes. FC, fold change.

Target genes corresponding to the nine active ingredients were retrieved from the TCMSP, SwissTargetPrediction, STITCH 5, SEA, Binding DB, and TargetNet databases. NASH-related targets were collected from DisGeNet, TTD, OMIM, Harmonizome, MalaCards, GeneCards, OpenTargets, and the GSE17470 dataset (Figure 1A). After removing non-protein-coding and repetitive genes, we identified 914 genes as potential *Aloe vera* targets (Supplementary Table S1) and 1078 genes as NASH-related targets (Supplementary Table S2). We overlapped the NASH and *Aloe vera*-related targets and obtained 260 *Aloe vera*–NASH common targets (Figure 1B, Supplementary Table S3). By tracing the common targets, we found that all nine *Aloe vera* active ingredients targeted multiple genes, suggesting that each of them could contribute to the anti-NASH effects of *Aloe vera* (Figure 1C). In addition, 96 targets interacted with at least two active ingredients, suggesting that the *Aloe vera* active ingredients exert synergistic effects by targeting similar genes (Figure 1D).

### 2.3. GO and KEGG Pathway Enrichment Analyses for Aloe vera–NASH Common Targets

**Figure 2:**
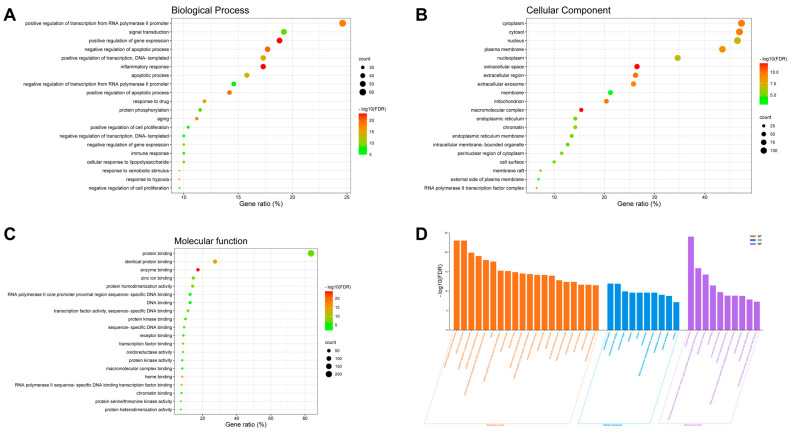
Gene Ontology (GO) enrichment analysis of *Aloe vera*–non-alcoholic-steatohepatitis (NASH) common targets. Bubble charts of top 20 GO terms based on gene ratio in the categories of (**A**) biological process, (**B**) cellular component, and (**C**) molecular function. The *X*-axis and *Y*-axis show gene ratios (%) and GO terms, respectively; the color and size of each bubble represent false discovery rate (FDR) values and gene count, respectively. (**D**) Top GO terms by FDR values. BP, biological process; CC, cellular component; MF, molecular function.

To elucidate the biological functions of the predicted target proteins, we performed GO and KEGG enrichment analyses of the 260 *Aloe vera*–NASH common targets using the DAVID database. The target proteins were highly enriched in 466 GO terms (Supplementary Table S4), many of which were related to transcriptional activity, such as the positive regulation of transcription from the RNA polymerase II promoter, RNA polymerase II transcription factor complex and activity, and transcription factor binding (Figure 2). Importantly, the targets showed close associations with the key histological and metabolic features involved in NASH pathology, including inflammation, glucose and cholesterol metabolism, apoptosis, and liver fibrosis (e.g., cellular response to lipopolysaccharide [16] and response to hypoxia [17]).

**Figure 3:**
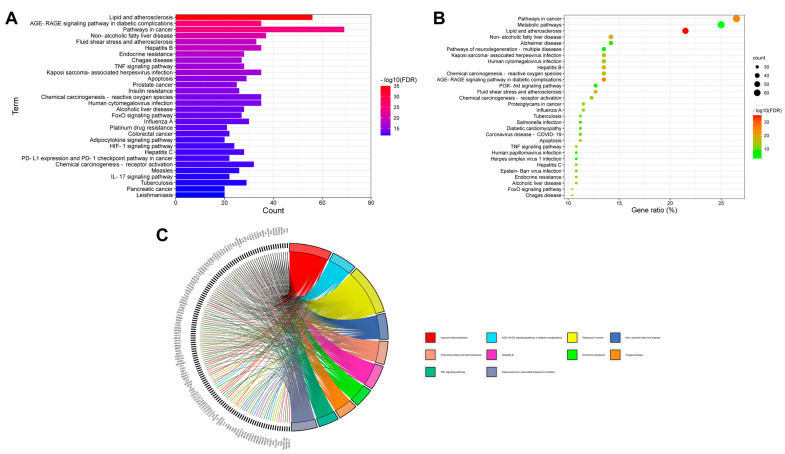
Kyoto Encyclopedia of Genes and Genomes (KEGG) pathway enrichment analysis of *Aloe vera*–NASH common targets. (**A**) Bar chart of the top 30 KEGG pathways by false discovery rate (FDR) values. (**B**) Bubble chart of the top 30 KEGG pathways by gene ratio (%). (**C**) Chord diagram of the top 10 KEGG pathways by FDR values and corresponding targets. NASH, non-alcoholic steatohepatitis.

The KEGG analysis showed 152 pathway enrichments (Supplementary Table S4); the top 30 pathways by FDR and gene ratio are illustrated in Figure 3. Previous studies have demonstrated the anti-inflammatory, anti-apoptotic, and antioxidant effects of *Aloe vera* on NASH in vivo [12,18], as well as its protective roles against atherosclerosis [19] and type 2 diabetes in humans [20]. Consistent with these studies’ findings, our results showed that the target pathways were mainly related to metabolic disorders (e.g., atherosclerosis, NAFLD, and insulin resistance), cell injury and death (e.g., apoptosis, TNF signaling [21], interleukin-17 signaling [22], programmed death-1 ligand 1 [PD-L1] expression, and the PD-1 checkpoint pathway [23]), oxidative stress (e.g., forkhead box O transcription factors [FOXO] signaling [24], phosphoinositide-3-kinase–protein kinase B/Akt [PI3K-AKT] signaling [25], and advanced glycation end product-receptor for advanced glycation end product [AGE-RAGE] signaling [26]), and liver fibrosis (e.g., TNF signaling, [27] adipocytokine signaling [28], and hypoxia-inducible factor 1 [HIF-1] signaling [29]). These results indicated that *Aloe vera*’s active ingredients modulated a wide range of pathological pathways and processes implicated in NASH. In addition, the “pathways in cancer” were highly enriched in the KEGG analysis, involving 69 (26.5%) out of the 260 common genes. Given that *Aloe vera* ingredients, such as aloe-emodin and aloin, have shown preventive and therapeutic effects against HCC via the modulation of apoptosis [30,31,32], we suspected that *Aloe vera* also exerts beneficial effects against NASH-associated HCC, although further studies are needed to validate this hypothesis. Additionally, several enriched pathways were associated with infectious diseases, such as hepatitis B and COVID-19, which have been linked to NASH [33]. NASH is increasingly observed in patients with chronic hepatitis B and worsens their prognoses [34].

### 2.4. C-T-P Network of Aloe vera–NASH Common Targets

**Figure 4:**
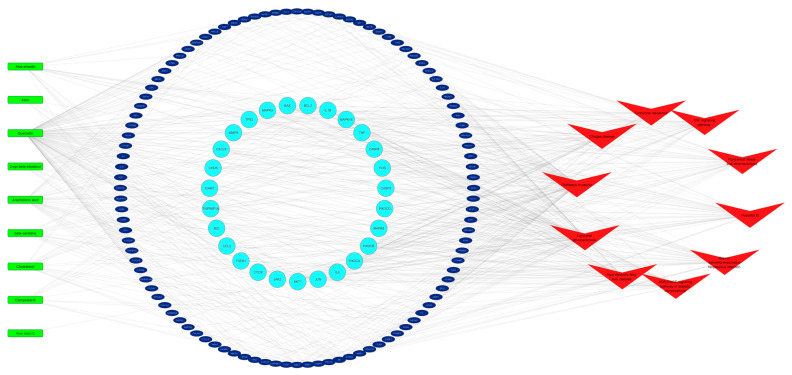
Compound–target–pathway network. Green and red nodes represent the active ingredients of *Aloe vera* and the top 10 KEGG pathways, respectively. Blue circles reflect the 27 genes involved in at least five pathways; navy ellipses show the remaining targets.

To decipher the targets that contribute significantly to the major pathways of *Aloe vera* against NASH, we collected 131 *Aloe vera*–NASH common targets that participated in the top 10 KEGG pathways based on FDR values (Table S5). A C–T–P network (Figure 4) was generated, which showed 27 proteins involved in at least five pathways; this indicated that these proteins were critical contributors to the multi-target and multi-pathway characteristics of *Aloe vera* for NASH treatment.

### 2.5. PPI Network of Aloe vera–NASH Common Targets

**Figure 5:**
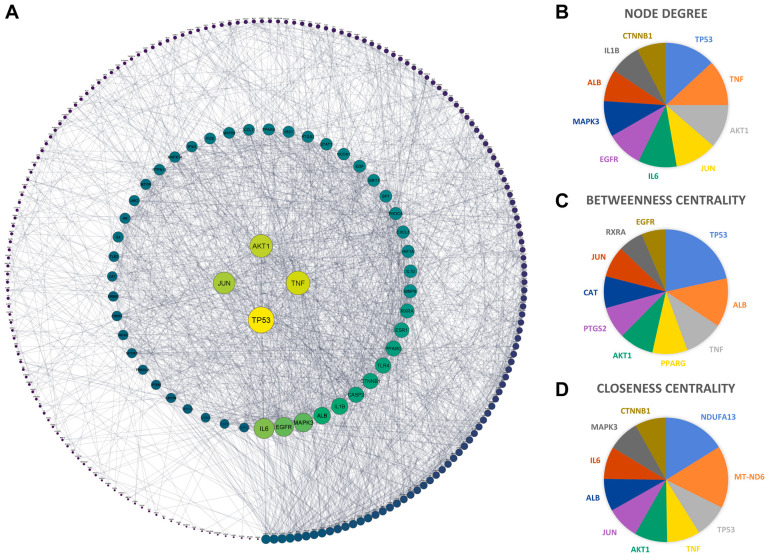
(**A**) Protein–protein interaction network of *Aloe vera*–NASH common targets. Nodes and edges represent the targets of *Aloe vera* against NASH and their interactions. Node size and color reflect degree value; the lower the degree value, the darker and smaller the node. Edge thickness represents the combined score from the STRING database. (**B**–**D**) Pie charts showing the top 10 genes with the highest values of (**B**) node degree, (**C**) betweenness centrality, and (**D**) closeness centrality.

To further identify the key proteins responsible for the anti-NASH action of *Aloe vera*, we established a PPI network of 260 common targets using the STRING database and Cytoscape 3.9.1. The PPI network included 252 nodes and 1901 edges, with an average node degree of 14.6 (Figure 5A). Next, we used the NetworkAnalyzer tool in Cytoscape to evaluate the topological properties of the nodes. The median values of node degree, betweenness centrality, and closeness centrality were 11, 0.0017, and 0.38, respectively. Generally, nodes with high topological properties are likely to play significant roles in the PPI network. In this study, we collected 10 genes with the highest values for each topological parameter for subsequent analyses (Figure 5B–D).

### 2.6. Core Targets of Aloe vera against NASH

**Figure 6:**
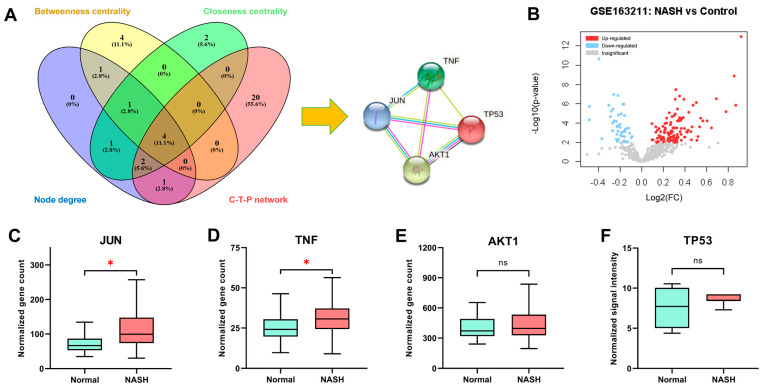
(**A**) Venn diagram of hub nodes of the protein–protein interaction network based on node degree (blue), betweenness centrality (yellow), closeness centrality (green), and hub nodes of the compound–target–pathway network (red). Four proteins that belong to every group, TP53, AKT1, TNF, and JUN, were considered the core targets of *Aloe vera* for NASH treatment. (**B**) Volcano plot of differentially expressed genes from the GSE163211 dataset. Red and blue dots represent upregulated and downregulated genes, respectively. Gray dots indicate insignificant differences. (**C**–**F**) Comparison of the mRNA expression of *JUN* (**C**), *TNF* (**D**), *AKT1* (**E**), and *TP53* (**F**) between normal (blue) and NASH (red) subjects. *JUN*, *TNF*, and *AKT1* mRNA levels were retrieved from the GSE163211 dataset, while *TP53* mRNA levels were extracted from the GSE17470 dataset. FC, fold-change; *, significant; ns, not significant.

By merging the hub nodes from the PPI network with those from the C–T–P network, we found that AKT1, TNF, JUN, and TP53 were present in all four groups, indicating that they were potentially the core targets of *Aloe vera* for NASH treatment (Figure 6A). Next, we studied the changes in the expression of these targets during NASH development. We performed DGE analysis of the GSE163211 (Figure 6B) and GSE17470 datasets to determine mRNA expression. The liver tissue of NASH patients exhibited increased *JUN* and *TNF* mRNA expression compared to normal individuals. In contrast, there was no significant difference in hepatic *AKT1* and *TP53* mRNA levels between the two cohorts (Figure 6C–F). *JUN*-encoded protein (c-Jun) expression is reported to be absent in healthy livers but expressed in hepatocyte nuclei in NASH livers [35,36]. Similarly, NASH patients display the upregulation of hepatic p53 (*TP53*-encoded protein) [37]. By contrast, AKT1 protein and phosphorylation at serine 473, which reflects its activation, decrease in NASH patients’ livers compared with the control group [38,39]. Data on the expression of TNFα (*TNF*-encoded protein) in NASH livers is limited; however, TNFα elevation has been documented in the livers of NASH mice [40] and the serum and adipose tissue of NASH patients [21]. All of the core targets showed altered protein or mRNA levels in NASH patients’ livers, indicating that they play critical roles in NASH onset and progression.

### 2.7. Molecular Docking Verification

The core proteins, including AKT1, c-Jun, TNFα, and p53, were used as receptors for molecular docking validation. Quercetin is a flavonol found in many plants and has been demonstrated to bind to AKT1, c-Jun, TNFα, and p53 [41,42]. Our study indicated the presence of quercetin in *Aloe vera*; therefore, it was chosen as the reference compound to assess the binding affinity between the other eight active ingredients and four core proteins of *Aloe vera*. Molecular docking results are shown in Table 2, which highlights that many active components could bind to the core proteins equally or better than the reference compound. Among the core targets, AKT1 is a protein kinase that plays an important role in controlling cell proliferation, differentiation, and survival [43]. Most of the active ingredients displayed strong binding affinities for AKT1; cholesterol and 3-epi-beta-sitosterol showed an affinity of −10.8 kcal/mol, while aloe-emodin and campesterol bound to AKT1 with docking score values of −10.2 and −11.0, respectively. The active ingredients also showed strong binding affinities for TNFα, with five of them having stronger docking scores than quercetin, including 3-epi-beta-sitosterol and campesterol (−8.7 kcal/mol), cholesterol (−8.5 kcal/mol), aloin (−8.3 kcal/mol), and aloe-emodin (−7.9 kcal/mol). These results suggest that AKT1 and TNFα may be the main targets of *Aloe vera* for NASH treatment.

Anthraquinone glycosides, which are a derivative of anthraquinones with an aglycone group, are derived from plants from several families. Studies in pharmacological activities show antiviral activity against HIV-1, as well as antioxidant, anti-inflammatory, and antidiabetic effects, with protective action against liver cirrhosis [44,45]. Among them, aloin is considered a well-known compound in this group. In previous studies, aloin showed the ability to fight chronic alcoholic liver injury via different mechanisms. Notably, aloin has been shown to play a role in NASH treatment in a mouse model via the activation of the nuclear factor erythroid 2-related factor/heme oxygenase-1 (Nrf2/HO-1) signaling pathway [46,47].

**Figure 7:**
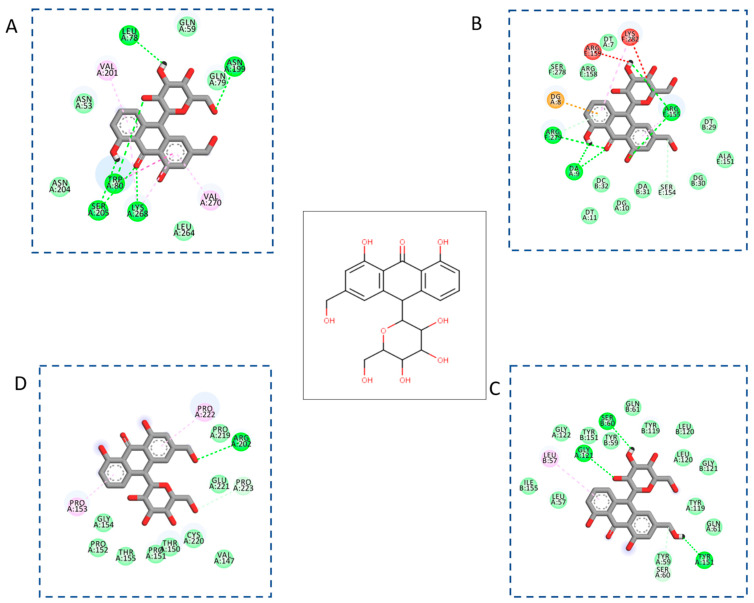
Interactions between aloin and the core targets. (**A**) AKT1, (**B**) c-Jun, (**C**) TNFα, and (**D**) p53.

Aloin is a major component derived from the inner sheath cells of *Aloe vera* leaves with a concentration of 0.9% dried leaf weight [48]. Our study indicated aloin has strong binding affinities for TNFα, AKT1, c-Jun, and p53 (Figure 7). Since the biological activities of NPs depend largely on the main ingredients, aloin may be a key contributor to the anti-NASH effects of *Aloe vera*.

## 3. Discussion

NASH is a pressing public health issue, placing a substantial burden on individuals and health services. The race to find therapeutics for NASH treatment is competitive. However, treatment options remain limited, mainly because the multi-factorial nature of the disease makes it difficult for one drug to address all of its pathological features. In this regard, NPs have attracted substantial attention. The major advantage of NASH treatment using NPs lies in their rich compositions, containing diverse compounds that simultaneously target multiple proteins and biological processes. Moreover, NPs are often associated with low cost and few side effects, critical factors in treating chronic diseases like NASH. *Aloe vera* has been used as a NP for thousands of years and has shown high economic returns for its multi-purpose applications in the food, cosmetic, and pharmaceutical industries.

**Figure 8:**
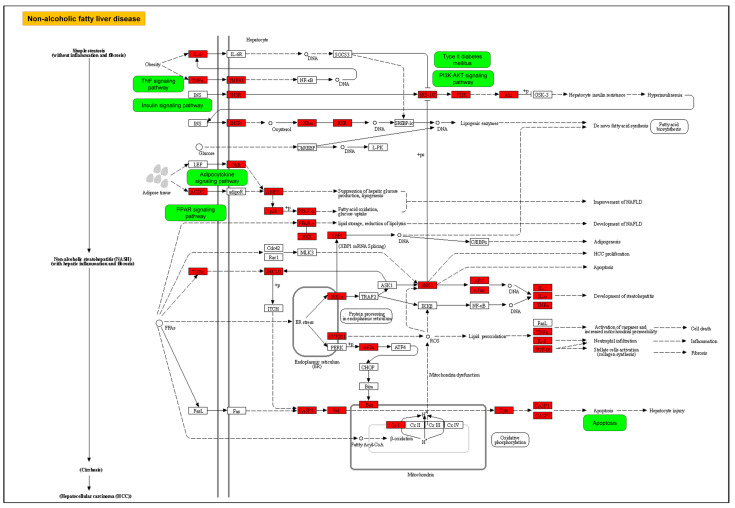
Pathway map of the mechanisms of *Aloe vera* against NASH. This figure was adapted from the reference pathway of NAFLD from the KEGG database (https://www.kegg.jp/pathway/map04932/, accessed on 2 September 2022). Target genes (red) and pathways (green) of *Aloe vera* for NASH treatment were obtained from a target fishing process and KEGG enrichment analysis, respectively. DNA, deoxyribonucleic acid; NASH, non-alcoholic steatohepatitis; NAFLD, non-alcoholic fatty liver disease; TNF, tumor necrosis factor.

Recent research reports the anti-NASH features of *Aloe vera* crude extract [12], but the molecular mechanisms remain unknown. This study aimed to explore the potential mechanisms underlying the therapeutic effects of *Aloe vera* against NASH through network pharmacology and molecular docking. The results suggest that the active ingredients of *Aloe vera* can target many key proteins associated with multiple pathological processes implicated in NASH (Figure 8). Among the predicted target proteins, AKT1 and TNFα are potentially the core targets of *Aloe vera* for NASH treatment, while c-Jun and p53 may also contribute to the anti-NASH features.

AKT1 belongs to the AKT kinase family and is a critical mediator of signal transduction pathways downstream of receptor tyrosine kinases and PI3K. The maximal activation of AKT1 requires two phosphorylation steps by PDK1 at Thr308 and mammalian target of rapamycin complex (mTORC) 2 at Ser473 [43]. Activated AKT1 transmits signals to downstream effectors to regulate various biological processes, including cell metabolism and survival [43]. In vivo research shows that AKT1 inhibition attenuates NAFLD development [49]. Herein, we reported that nine active *Aloe vera* ingredients formed stable bindings with AKT1. Moreover, some of these ingredients have previously been reported to reduce AKT phosphorylation, including quercetin [50] and aloin [51], suggesting that the inhibitory effect of *Aloe vera* on NASH is partly mediated by the inactivation of AKT1. A possible mechanism is that active compound bindings hinder AKT1 phosphorylation sites or change its conformation, thus preventing its phosphorylation by the upstream activators.

TNFα is a pro-inflammatory cytokine that plays critical functions in regulating the immune system. Emerging evidence shows the involvement of TNF signaling in NASH progression, and the blockage of this pathway leads to the resolution of NASH [21]. *Aloe vera* administration has been shown to reduce TNFα levels [52]. Herein, molecular docking indicated that many components of *Aloe vera* might directly interact with TNFα, suggesting that the bindings with the active compounds may destabilize TNFα, shorten its half-life, or suppress its production.

**Figure 9:**
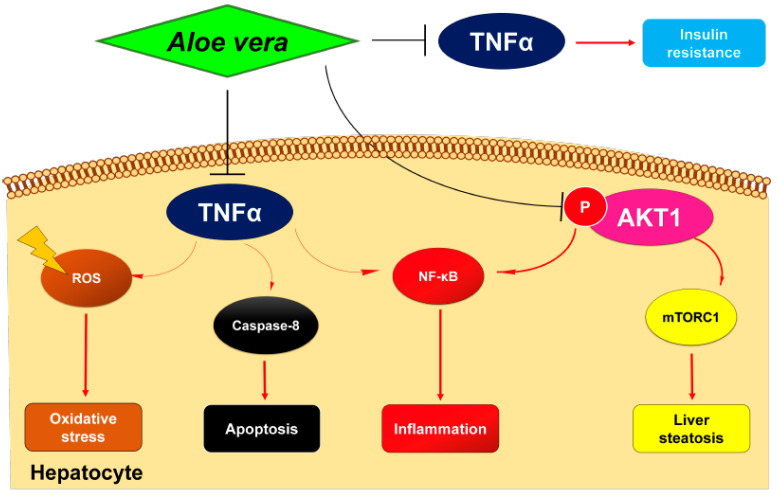
Schematic diagram of *Aloe vera* core targets for NASH treatment. AKT1, AKT serine/threonine kinase 1; mTORC1, mammalian target of rapamycin complex 1; NF-κB, nuclear factor-kappa B (NF-κB) ROS, reactive oxygen species; TNFα, tumor necrosis factor alpha.

In addition, this study demonstrated that *Aloe vera* exerts therapeutical effects on NASH by affecting glycolipid metabolism, inflammation, apoptosis, oxidative stress, liver fibrosis, and possibly NASH-associated HCC by targeting the core proteins. Figure 9 illustrates some mechanisms proposed for this activity

Lipid metabolism. Upon activation, AKT1 promotes the synthesis of fatty acids and lipids within hepatocytes via mTORC1/SREBP1 signaling [53]. As a result, the overexpression of AKT1 triggers liver steatosis [54], while AKT1 inactivation relieves hepatic lipid abnormalities [55]; this suggests that AKT1 suppression may mediate the anti-steatotic effect of *Aloe vera* against NASH. Additionally, AKT1 inhibition ameliorates hyperlipidemia, a common metabolic complication in NASH patients [56]. Therefore, *Aloe vera* treatment may potentially attenuate the development of cardiovascular disease, which is the primary cause of mortality in NASH patients.

Glucose metabolism. Insulin resistance is central to the pathogenesis of NASH. Adipose tissue insulin resistance leads to sustained lipolysis, which increases circulating fatty acid and ultimately impairs whole-body insulin signaling [26]. Therefore, therapies for NASH also need to address insulin resistance. Previous studies report that circulating TNFα interferes with insulin signaling through its receptor [57], while TNFα knockout mitigates insulin resistance in obese mice [58]. Therefore, TNFα may be the key contributor to the antidiabetic effect of *Aloe vera* in NASH treatment.

Inflammation. Nuclear factor-kappa B (NF-κB) is a family of transcription factors coordinating immune development and immune and inflammatory responses [59]. Inactivated NF-κB exists in the cytosol complexed with the inhibitor of factor kappa B (IκB) [59]. AKT1 and TNFα promote the phosphorylation and degradation of IκB, resulting in the release and nuclear translocation of NF-κB [60], which in turn binds to specific deoxyribonucleic acid (DNA) sequences to regulate the transcription of pro-inflammatory proteins, including TNFα and IL6 [59]. Conversely, the inhibition of AKT1 or TNFα maintains NF-κB–IκB association, thus repressing NASH inflammation [61,62]. Therefore, the anti-inflammatory feature of *Aloe vera* may be the combined effect of multiple targets.

Apoptosis. The antitumor characteristic of *Aloe vera* is often associated with apoptosis induction [30,31,32]. However, hepatocyte apoptosis drives liver injury, inflammation, and fibrosis during NASH progression [63], so the induction of the apoptotic process may seem to be a side effect of *Aloe vera* in NASH treatment. Unexpectedly, *Aloe vera* crude extract shows an inhibitory effect on apoptosis in NASH rats [12]; this may be explained by the differences in the composition of *Aloe vera* extracts among studies. Xu et al. (2021) verified that aloin inhibits apoptosis in NASH mice via the activation of the Nrf2/HO-1 pathway, which has been shown to reduce TNFα production [64]. Given that TNFα can trigger apoptosis via caspase-8 activation [65], it is likely that aloin-mediated TNFα inhibition plays a critical role in the anti-apoptotic effect of *Aloe vera* against NASH.

Oxidative stress. Oxidative stress triggers necroinflammation and apoptosis during NASH, while antioxidants, such as vitamin E, confer benefits on NASH patients [66]. The antioxidant effect of *Aloe vera* is well-established and has also been documented in NASH rats [12]. *Aloe vera* treatment downregulates TNFα [67], which induces the production of reactive oxygen species in mitochondria during inflammation [68]. Therefore, TNFα inhibition may contribute to *Aloe vera*’s antioxidant effect in treating NASH.

Liver fibrosis. Fibrosis severity is the strongest prognostic predictor for the long-term outcomes of NASH [69]. Previous studies have reported the protective effects of *Aloe vera* treatment against viral infection and chemical-induced liver fibrosis [52,70]. Additionally, TNFα inhibitors have shown promising efficacy in alleviating fibrosis in patients with NASH [21]. While *Aloe vera*’s benefits for treating NASH-related fibrosis has not been confirmed, current evidence supports the idea that it may exert beneficial effects on fibrotic NASH.

Hepatocellular carcinoma. AKT1 acts as an oncogene across many tumor types, including HCC. The hyperactivation of AKT1 signaling predicts poor prognosis in patients with HCC [71], while AKT1 inhibition suppresses HCC proliferation [72,73]. Many studies have shown that *Aloe vera* and its active components, including aloe-emodin and aloin, attenuate HCC progression [30,31,32]; therefore, *Aloe vera* treatment may potentially be beneficial for the treatment of NASH-associated HCC.

NPs’ biological activities are largely dependent on their main ingredients. Aloin is a major component of *Aloe vera*’s leaves and has confirmed anti-NASH effects in vivo [15]. Moreover, aloin has been shown to reduce AKT1 phosphorylation and TNFα content in the liver [15,74], suggesting that aloin may be a prime ingredient for the inhibitory effects of *Aloe vera* on NASH. However, the safety of aloin treatment is of concern; an overdose causes severe abdominal cramps, diarrhea, potassium depletion, and volume depletion [75]. Nonetheless, the therapeutic potentials of low-dose aloin should not be ignored. Besides, aloin can serve as a hit compound for designing new drugs with better safety and tolerance profiles. This study’s results suggest that *Aloe vera* and its active ingredients are potentially useful for NASH treatment. Despite a lack of experimental verification, the conclusions were consistent with those of previous research studies. Our findings provide a direction for future research on *Aloe vera*’s potential benefits for treating and managing NASH and associated diseases, such as HCC.

## 4. Materials and Methods

### 4.1. Screening of the Active Ingredients of Aloe vera

The *Aloe vera* ingredients were collected from the Traditional Chinese Medicine System Pharmacology (TCMSP, Version 2.3. https://tcmspw.com/tcmsp.php, accessed on 2 September 2022) using the keyword “Aloe” [76]. Oral bioavailability (OB) represents the extent and rate at which a compound is absorbed in the systemic circulation; drug-likeness (DL) compares a compound’s physical and biochemical properties with those of known drugs to predict the chance for it to become a drug candidate. High OB and DL values are good indicators of a biologically active molecule. This study selected compounds with an OB ≥30% and DL ≥0.18 [77]. Additionally, we added aloin, an extract derived from *Aloe vera* leaves, to the active ingredients since this compound has shown anti-NASH features in animal research [15]. Besides, aloin is also the main ingredient in different parts of *Aloe vera*. Therefore, the activity of the extract is predicted to be similar to that of the dominant components. The PubChem database (https://pubchem.ncbi.nlm.nih.gov, accessed on 2 September 2022) was employed to obtain the collected compounds’ standard names, SMILES, and three-dimensional (3D) structures for subsequent target prediction.

### 4.2. Prediction of Aloe vera and NASH-Related Targets

Putative targets of the active ingredients of *Aloe vera* were retrieved from the TCMSP [76], SwissTargetPrediction [78], Search Tool for Interacting Chemicals (STITCH 5) [79], Similarity Ensemble Approach (SEA) [80], BindingDB [81], and TargetNet [82] databases. NASH-related targets were acquired from DisGeNet [83], Online Mendelian Inheritance in Man (OMIM) [84], MalaCards [85], Harmonizome [86], Therapeutic Target Database (TTD) [87], OpenTargets [88], and GeneCards [89] databases. We also performed differential gene expression (DGE) analysis on the GSE17470 dataset (GPL2895 platform) [90], which contains the global gene expression of seven NASH patients and four normal controls, using the limma package in R Version 4.2.1 [91]. Detailed selection criteria are shown in Table 3. The collected genes were imported into the UniProt database to obtain their official names for “Homo sapiens” and corresponding UniProt IDs. By removing non-protein-coding, non-“Homo sapiens,” and duplicate genes, *Aloe vera-* and NASH-related targets were obtained. A Venn diagram of *Aloe vera*–NASH’s common targets was plotted by the online Weishengxin bioinformatics (https://www.bioinformatics.com.cn/, accessed on 2 September 2022) data analysis and visualization platform, and a network of herb–component–target-disease was generated using Cytoscape Version 3.9.1 [92].

### 4.3. Gene Ontology and Kyoto Encyclopedia of Genes and Genomes Pathway Enrichment Analyses

Gene Ontology (GO) and Kyoto Encyclopedia of Genes and Genomes (KEGG) enrichment analyses of *Aloe vera*–NASH common targets were performed using the *Database* for Annotation, Visualization and Integrated Discovery (DAVID) 2021 database [93]. “Homo sapiens” was selected as the organism; a FDR < 0.01 was considered statistically significant. The top GO terms and KEGG pathways by gene ratio and FDR were plotted using the Weishengxin bioinformatics (http://www.bio-informatics.com.cn/, accessed on 2 September 2022) data analysis and visualization platform.

### 4.4. Construction of the Compound–Target–Pathway Network

We extracted *Aloe vera*–NASH common targets involved in the top 10 KEGG pathways by FDR values from DAVID 2021 database. Then, a compound–target–pathway (C–T–P) network was established using Cytoscape Version 3.9.1. Proteins that participated in at least five pathways were considered hub nodes of the C–T–P network.

### 4.5. Construction of Protein–Protein Interaction Network

*Aloe vera*–NASH common targets were uploaded into the STRING Version 11.5 database [94]. “Homo sapiens” was selected; the minimum required interaction score was set at 0.700, and disconnected proteins were excluded.

The obtained data were imported to Cytoscape version 3.9.1 for visualization. Three topological parameters, including degree, betweenness centrality, and closeness centrality, were determined using the NetworkAnalyzer tool [95] in Cytoscape, wherein “degree” was the number of direct links to a node, “betweenness centrality” reflected how frequently the shortest paths between all pairs of nodes pass through a node, and “closeness centrality” determined the mean length of the shortest paths that connect a node to others. Proteins with the highest topological parameter values were considered hub nodes of the protein–protein interaction (PPI) network.

### 4.6. Identification of Aloe vera’s Core Targets against NASH

The hub nodes from the C–T–P and PPI networks were compared, and overlapping nodes were identified as *Aloe vera*’s core targets against NASH. The Venny 2.1.0 [96] online tool was used for visualization. Changes in messenger ribonucleic acid (mRNA) and the protein expression of the core targets in the context of NASH were obtained through DGE analysis of the GSE163211 dataset (GPL29503 platform) and a previous publication, respectively [97].

The GSE163211 database housed the mRNA expression of 795 genes with known or presumed relevance to liver steatosis, inflammation, and fibrosis from 154 NASH samples, 82 with and 72 without fibrosis, of which 76 were normal obese samples. Consequently, this study overcame several major weaknesses of other studies, such as small sample sizes, few advanced NASH tissue samples, and a lack of controls with similar risk factors to the human disease. Since the genes in this dataset were not randomly chosen, *p* < 0.01 was used as the indicator of statistical significance [97]. Additionally, the *TP53* gene was not included in GSE164760, so we used the GSE17470 dataset for this gene. GraphPad Prism (Version 8.0.0; GraphPad Software, San Diego, CA, USA, www.graphpad.com, accessed on 2 September 2022) was applied for data visualization.

### 4.7. Molecular Docking Simulation

Protein and ligand preparation. Four predicted core targets of *Aloe vera* for NASH were selected for the simulation. The 3D crystal structures of the proteins, including AKT serine/threonine kinase 1 (AKT1), transcription factor c-Jun (c-Jun), tumor necrosis factor alpha (TNFα), and tumor protein p53 (p53) (PDB ID: 3O96, 1FOS, 2AZ5, 3ZME, respectively), were downloaded from the Protein Data Bank. Existing ligand and water molecules were removed using Biovia Discovery Studio 2020 visualizer (Version 2020; BIOVIA, Dassault Systèmes, San Diego, CA, USA). Protein structures were prepared using AutoDock Tool Version 1.5.6, including the steps of “add hydrogens,” “add Kollman charges,” and “exported into a dockable pdbqt format for molecular docking.” The 3D structures of active compounds of *Aloe vera* were downloaded from the PubChem database, and all ligands were converted to dockable pdbqt format using Open Babel 3.1.1.

Molecular docking. Molecular docking of the bioactive compounds on target proteins was conducted using AutoDock Vina 1.1.2. Docking scores were reported in kcal/mol. Finally, molecular interactions between the proteins and ligands were visualized by Biovia Discovery Studio Visualizer.

## 5. Conclusions

This study shows that *Aloe vera*’s active ingredients affect several pathological processes implicated in NASH, including inflammation, glucose and lipid metabolism, apoptosis, oxidative stress, and fibrosis through multiple pathways, such as TNF and PI3K-AKT signaling. Molecular docking results indicate that the active ingredients interact directly with several proteins involved in NASH pathology, including AKT1, TNFα, c-Jun, and p53. This study’s results support to develop *Aloe vera*-based NPs as complementary treatments for NASH. Furthermore, it identifies novel drug candidates based on the structures of *Aloe vera*’s active compounds. However, in vitro and in vivo studies are needed to evaluate the activity and safety in the use of *Aloe vera* and its components in the treatment of NASH.

## Figures and Tables

**Table 1:** Active ingredients of *Aloe vera*.

Molecule ID	Molecule Name	PubChem CID	OB (%)	DL
MOL001439	Arachidonic acid	444899	45.57	0.20
MOL002773	Beta-carotene	5280489	37.18	0.58
MOL000359	3-Epi-beta-sitosterol	12303645	36.91	0.87
MOL000471	Aloe-emodin	10207	83.38	0.24
MOL005043	Campesterol	173183	37.58	0.71
MOL005051	Aloe resin C	11972360	34.99	0.50
MOL000953	Cholesterol	5997	37.87	0.68
MOL000098	Quercetin	5280343	46.43	0.28
MOL005060	Aloin	14989	22.18	0.71

**Table 2:** Binding affinities between active components and core targets of *Aloe vera*.

No.	Compound	Docking Score (kcal/mol)			
		AKT1	TNFα	c-JUN	p53
1	Arachidonic acid	−7.1	−6.3	−7.2	−5.9
2	Beta-carotene	−8.7	−7.4	−3.9	−5.1
3	3-Epi-beta-sitosterol	−10.8	−8.7	−5.6	−6.1
4	Aloe-emodin	−10.2	−7.9	−10.1	−7.8
5	Campesterol	−11.0	−8.7	−4.5	−5.0
6	Aloe resin C	−9.3	−7.6	−6.3	−6.1
7	Cholesterol	−10.8	−8.5	−4.6	−5.2
8	Aloin	−8.9	−8.3	−9.5	−5.7
9	Quercetin (reference)	−9.7	−7.7	−10.5	−8.1

**Table 3:** Data sources and selection criteria for target fishing.

No	Data Source	Keyword	Selection Criteria
***Aloe vera*-Related Targets**			
1	TCMSP	Aloe	
2	SwissTargetPrediction	SMILES structures of the active ingredients	Probability ≥ 0.1
3	STITCH 5		Interaction score ≥ 0.400
4	SEA		MaxTC > 0.28, *p*-value < 0.05
5	Binding DB		Similarity ≥ 0.7
6	TargetNet		Score ≥ 0.8
**NASH-Related Targets**			
1	DisGeNet	Non-alcoholic steatohepatitis	
2	OMIM		
3	MalaCards		
4	Harmonizome		
5	Therapeutic Target Database	Non-alcoholic steatohepatitis	
6	OpenTargets		Overall association score ≥ 0.1
7	GeneCards	(Non-alcoholic steatohepatitis) OR (Non-alcoholic steatohepatitis)	Protein-coding genes
8	GSE17470 dataset		|log_2_(fold-change)| > 2; false discovery rate (FDR) < 0.01

## Supplementary Materials

The following supporting information can be downloaded at: https://www.mdpi.com/article/10.3390/plants11243585/s1, Table S1: Targets of *Aloe vera*; Table S2: NASH-related targets; Table S3: Common targets of *Aloe vera* and NASH; Table S4: GO and KEGG pathway enrichment analyses; Table S5: Targets in the compound–target–pathway map.
